# The Human Cytomegalovirus Assembly Compartment: A Masterpiece of Viral Manipulation of Cellular Processes That Facilitates Assembly and Egress

**DOI:** 10.1371/journal.ppat.1002878

**Published:** 2012-09-20

**Authors:** James C. Alwine

**Affiliations:** Department of Cancer Biology, School of Medicine, University of Pennsylvania, Philadelphia, Pennsylvania, United States of America; University of Florida, United States of America

## The Human Cytomegalovirus Assembly Compartment Results from Extensive Reorganization of Secretory Organelles and Other Cellular Components in Order to Form a Juxtanuclear Body That Facilitates Assembly and Egress of Virions

**Figure 1:**
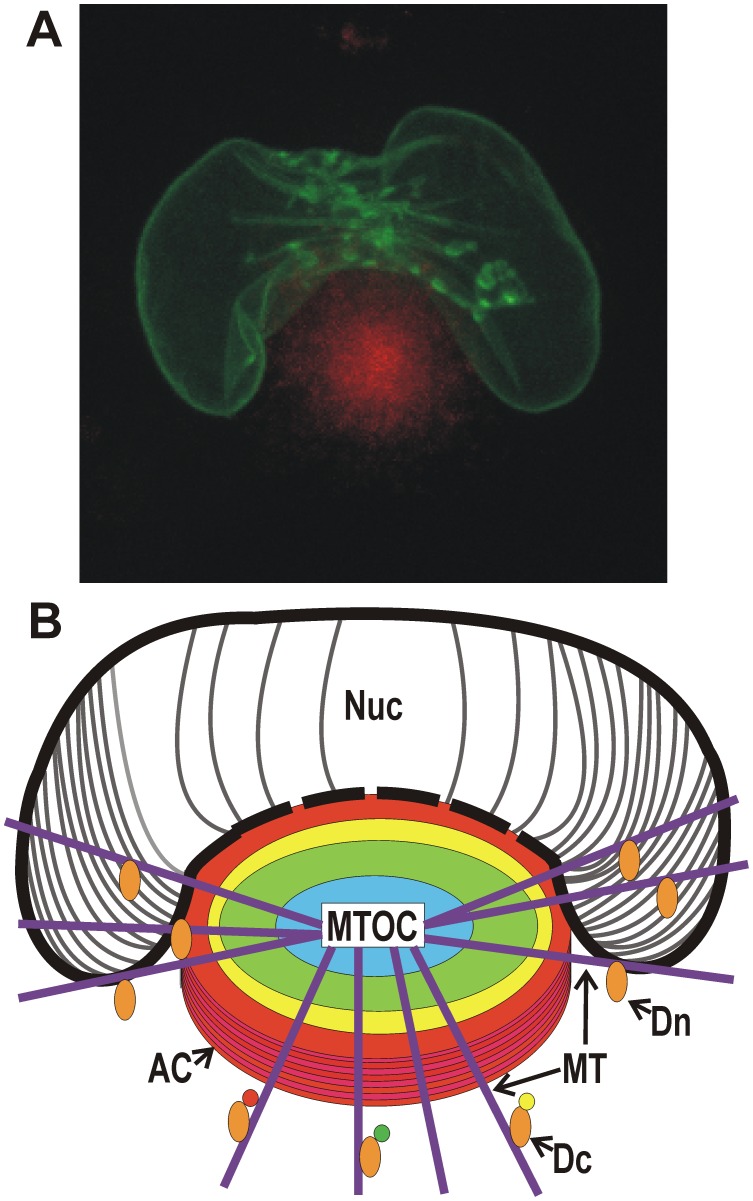
Microscopic and diagrammatic representations of the assembly compartment and nucleus in an HCMV-infected cell. (A) Maximum projection live cell micrograph showing the AC (identified by Dsred-tagged pp28, red) with the enlarged, kidney-shaped nucleus (identified by GFP-tagged lamin A) wrapped around the AC. (B) A poor artist's diagrammatic representation of the AC located next to the enlarged, kidney-shaped nucleus. The AC is formed on a microtubule organizing center (MTOC) with microtubules (MT) radiating from it. The nested cylindrical makeup of the AC is indicated by colored circles; each cylindrical region is proposed to be made up of vesicles derived from specific secretory organelles. Virion tegument and structural proteins reside with these vesicles and are applied to nucleocapsids as they egress from the nucleus and traverse toward the center of the AC. The point of contact between the AC and the nucleus may be a continuum, suggested by the dashed line representing the nuclear membrane at this junction. This may allow nucleocapsids open access from the nucleus to the AC late in infection. The AC and the kidney-shaped nucleus are formed by virally commandeered functions of dynein. Dynein (Dn) is shown pulling the nucleus around the AC by a mechanism similar to mitotic nuclear envelope breakdown. Dynein loaded with cargo (Dc) is shown to represent the formation of the AC.

A characteristic feature of human cytomegalovirus (HCMV) infected cells is an enlarged, kidney-shaped nucleus wrapping around a juxtanuclear body (also referred to as a perinuclear body) called the viral cytoplasmic assembly compartment (AC) (Figure 1A). This association of the nucleus and AC is vital for virion assembly and egress. The AC was first described [1], [2] while examining the localization of HCMV tegument proteins, which are incorporated into the virion. It was found that they localized to a juxtanuclear compartment that overlapped the endoplasmic reticulum-Golgi intermediate compartment. Detailed studies by Pellet and coworkers [3]–[5] have shown that the AC results from extensive remodeling of the secretory apparatus. From these data a three-dimensional model was proposed of a cylindrical AC composed of many organelle-specific vesicles (Golgi, trans-Golgi network, and early endosomes), which form nested, cylindrical layers (depicted by the colored circles in Figure 1B).

More recently, additional cellular components have been found associated with the AC, including markers of the late endocytic pathway, lysosomal markers, SNARE family members [6], [7], and the ER chaperone glucose-regulated protein 78 (GRP78) also known as BiP [8], [9]. BiP appears to play a role in AC stability; BiP depletion causes rapid AC disintegration and the cessation of infectious virion formation [9]. In addition, components of the ESCRT (endosomal sorting complex required for transport) machinery may associate with the AC. Since ESCRT controls the incorporation of cargo into intraluminal vesicles of multivesicular bodies, this association suggests that the ESCRT machinery may facilitate formation of the multivesicular AC and virion maturation [4]. Recent evidence shows that the AC also contains mammalian target of rapamycin (mTOR) kinase [10]. During infection HCMV strives to maintain mTOR kinase activity due to its importance in maintaining cellular processes needed by the virus. The data suggest that sequestration of mTOR and its activator Rheb-GTP in the AC helps maintain mTOR activity and protect mTOR from inhibition by cellular stress responses induced during lytic infection [10]. The AC also contains HCMV-encoded Fc receptor-like proteins which have an affinity for rabbit immunoglobulin G (IgG) [11], which can cause spurious localization of IgG to the assembly compartment. Thus when studying the AC by immunofluorescence it is best to use nonrabbit antibodies, or block with HCMV-negative human serum, which effectively binds the Fc receptors [9], [12]. The purpose of the Fc receptor-like proteins in the AC is unknown. A complete list of known cellular components of the AC, and how their function may be altered during infection, can be found in [4]. This compilation of cellular components in the AC is based on static imaging, thus dynamic partitioning of other cellular proteins is quite likely. Overall, the data show that the HCMV-induced remodeling of the membrane transport apparatus is extensive, altering the organization of secretory organelles in order to facilitate assembly and egress of virions.

## The AC Is a Milieu for Ordered Addition of Structural Proteins to the Nucleocapid for Virion Maturation

In addition to cellular components, the AC contains many viral structural and tegument proteins that are necessary for mature virion formation. As discussed in the following section a current model of AC structure and function suggests that each cylindrical layer of secretory-derived vesicles contains specific sets of tegument proteins that are transferred to nucleocapsids after they egress from the nucleus and move through the AC, and then proceed to envelopment and release [5], [13]–[15]. Thus by commandeering the vesicular secretary components to form the AC, HCMV creates a milieu where tegument proteins can be arranged for ordered application to the maturing virions, which can then be enveloped.

## Formation of the AC is Accompanied by Remodeling of the Nucleus, Including Increased Size, Altered Shape and Reorganization of the Nuclear Membrane and Nuclear Lamina to Facilitate Nucleocapsid Egress into the AC

The formation of the AC is accompanied by considerable nuclear remodeling; both must be considered with respect to the current model for herpesvirus assembly and egress. Nucleocapsids are formed in the nucleus and are believed to egress to the cytoplasm by acquiring an envelope while budding through the inner nuclear membrane (INM) [14], [16], [17]. These enveloped nucleocapsids move into the lumen of the nuclear envelope, the perinuclear space, where the INM-derived envelope fuses with the outer nuclear membrane (OMN), releasing naked nucleocapsids into the cytoplasm [13], [17]. As discussed above, the cytoplasmic nucleocapsids become mature virions by passage through the AC, followed by envelopment (reviewed in [18]). It is believed that nucleocapsids are actively transported through these processes, possibly utilizing molecular motors and the microtubules that radiate from a microtubule organizing center (MTOC) on which the AC is formed [5] (Figure 1B).

At least two events facilitate movement of nucleocapsids from the nucleus to the cytoplasm. First, the nuclear lamina, a network of proteins located on the nucleoplasmic side of the inner nuclear membrane, is reorganized to allow nucleocapsid access to the INM. This is accomplished by HCMV proteins UL50 and UL53, which form a complex with other viral and cellular proteins to phosphorylate the lamins and rearrange the lamina [19]–[21]. Studies have shown that the nuclear lamina adjacent to the AC is highly rearranged, presumably to allow nucleocapsids access to the nuclear membrane adjacent to the AC [21]; directional egress toward the AC has been suggested [8]. Thus a mechanism for nuclear sensing of the position of the AC may induce AC proximal changes in the nucleus that facilitate directional nucleocapsid egress.

A second event facilitating nucleocapsid egress from the nucleus involves nuclear membrane localized proteins, including the Sad1/UNC-84 homology (SUN) and Klarsicht, Anc-1, Syne homology (KASH) domain-containing proteins. SUN domain proteins form homo- and heterodimers; the N-termini cross the INM and anchor in the nuclear lamina. The C-terminal domains of the SUN proteins are located in the perinuclear space, where they interact with the KASH domains of the nesprin family proteins (reviewed in [22] and [23]), which associate with the ONM. The association of SUN and KASH domain proteins in the perinuclear space keeps the INM and ONM tethered and close together. The loss of SUN domain proteins results in increased space between the INM and ONM [24]; this occurs in HCMV-infected cells due a dramatic loss of SUN domain proteins [21]. The resulting destabilization of the nuclear membrane may aid nucleocapsid egress [21]. Indeed, several lines of evidence suggest that at late times of infection, when nucleocapsid maturation is greatest, the nuclear membrane next to the AC is not intact [21]. This suggests that there is a continuum between the nucleus and the AC, potentially allowing free flow of nucleocapsids into the AC.

## HCMV Commandeers Functions of the Molecular Motor Dynein for Formation of the AC and Reshaping the Nucleus around the AC

Clearly there is an intimate relationship between the concave nucleus and the AC. Formation of the AC is closely linked to the enlargement and remodeling of the nucleus, which wraps around the AC at late times in infection (Figure 1A). A key to understanding AC formation and nuclear remodeling was the observation that the AC forms on a MTOC and microtubules (MT) (Figure 1B) radiate out from the AC [1]. It has been shown that dynein is integral to AC formation [21], [25] as well as formation of the characteristic large, kidney-shaped nucleus seen in HCMV-infected cells [21]. Dynein is the predominant minus-end directed microtubule motor in eukaryotic cells; it functions with a second complex, dynactin, which acts as an adaptor that allows dynein to bind its cargo. Since dynein moves on microtubules toward the nucleus, it is reasonable to suggest that dynein's role in nuclear reshaping and AC formation is facilitated by microtubules radiating from the MTOC within the AC (Figure 1B). Studies of nuclear envelope breakdown (NEBD) during M-phase suggest that dynein attaches to the nuclear membrane and moves toward the centromere, generating tension on the nucleus and creating folds in the nuclear envelope [26], [27]. HCMV-infected cells appear to use a modified version of NEBD in which dynein binds AC components and the nuclear membrane and moves toward the MTOC that will form the center of the AC. This pulling of the nuclear membrane toward the MTOC can account for the concave nuclear shape around the AC and may promote tight association between the AC and the nucleus. During normal NEBD, the stress of dynein's pulling results in tearing of the nuclear membrane and nuclear breakdown. In infected cells, nuclear breakdown is apparently alleviated by nuclear enlargement, which relieves the stress by adding additional nuclear membrane; this may be one reason why increased fatty-acid synthesis is vital for HCMV infection [28].

## HCMV Does Not Create Novel Cellular Processes but Advantageously Manipulates Existing Cellular Mechanisms

While the formation of the AC and the accompanying remodeling of the nucleus appears to be a massive and irreparable abrogation of cell morphology, this is not the case. Perturbing the juxtanuclear position of the AC, or interfering with AC integrity, results in the nucleus regaining its normal shape and size [9], [19], [21], [29], [30]. Likewise, limiting the ability to alter the shape of the nucleus inhibits AC formation. Interestingly, these same conditions often abolish all microscopically apparent cytoplasmic viral activity, and the infected cells look relatively normal [8]. Thus the dramatic viral-mediated morphological alterations of the cell are reversible if the viral effects on cellular mechanisms are disrupted. This suggests, once again, that HCMV does not create new cellular processes or new biochemistry. Instead it has evolved elegant means to commandeer and manipulate what is available in the cell. In this case the virus has tapped into normal dynein-mediated functions for nuclear modeling and vesicular transport and used these to its advantage for remodeling the nucleus, forming the AC, and setting up an integrated nuclear–cytoplasmic assembly–egress continuum.
